# Neuroprotective Effects of Pomegranate Peel Extract after Chronic Infusion with Amyloid-β Peptide in Mice

**DOI:** 10.1371/journal.pone.0166123

**Published:** 2016-11-09

**Authors:** Maressa Caldeira Morzelle, Jocelem Mastrodi Salgado, Milena Telles, Danilo Mourelle, Patricia Bachiega, Hudson Sousa Buck, Tania Araujo Viel

**Affiliations:** 1 Department of Agri-food Industry, Food and Nutrition, ‘Luiz de Queiroz’ College of Agriculture, University of São Paulo, Pádua Dias Avenue, P.O. Box. 9, 13418–900, Piracicaba, SP, Brazil; 2 Graduation Course on Pharmacology, Institute of Biomedical Sciences, Universidade de São Paulo, Avenida Professor Lineu Prestes, 1524, 05508–900, São Paulo, Brazil; 3 Department of Physiological Sciences, Santa Casa de São Paulo School of Medical Sciences, R. Dr. Cesario Motta Junior, 61, 111° andar, São Paulo, SP 01221–020, Brazil; 4 School of Arts, Sciences and Humanities, Universidade de São Paulo, Av. Arlindo Bettio, 1000, São Paulo, SP 03828–080, Brazil; Torrey Pines Institute for Molecular Studies, UNITED STATES

## Abstract

Alzheimer’s disease is a chronic and degenerative condition that had no treatment until recently. The current therapeutic strategies reduce progression of the disease but are expensive and commonly cause side effects that are uncomfortable for treated patients. Functional foods to prevent and/or treat many conditions, including neurodegenerative diseases, represent a promising field of study currently gaining attention. To this end, here we demonstrate the effects of pomegranate (*Punica granatum*) peel extract (PPE) regarding spatial memory, biomarkers of neuroplasticity, oxidative stress and inflammation in a mouse model of neurodegeneration. Male C57Bl/6 mice were chronically infused for 35 days with amyloid-β peptide 1–42 (Aβ) or vehicle (control) using mini-osmotic pumps. Another group, also infused with Aβ, was treated with PPE (*p*.*o*.*–* βA+PPE, 800 mg/kg/day). Spatial memory was evaluated in the Barnes maze. Animals treated with PPE and in the control group exhibited a reduction in failure to find the escape box, a finding that was not observed in the Aβ group. The consumption of PPE reduced amyloid plaque density, increased the expression of neurotrophin BDNF and reduced the activity of acetylcholinesterase enzyme. A reduction in lipid peroxidation and in the concentration of the pro-inflammatory cytokine TNF-α was also observed in the PPE group. No hepatic lesions were observed in animals treated with PPE. In conclusion, administration of pomegranate peel extract has neuroprotective effects involving multiple mechanisms to prevent establishment and progression of the neurodegenerative process induced by infusion with amyloid-β peptide in mice.

## Introduction

Alzheimer's disease (AD) is a neurodegenerative condition related to the aging process with no cure until recently. The current interest in the benefits to human health of antioxidant compounds of fruits has led to many research projects in the field of AD [1].

The pomegranate (Punica granatum) is a rich source of bioactive compounds with antioxidant capacity [2]. In recent years, studies have shown that this fruit has numerous benefits to human health, which has increased interest in studying its therapeutic properties [3]. In mouse models of AD, the consumption of a diet rich in pomegranate pulp or the direct consumption of the fruit's juice increased behavioral performance and reduced amyloid plaque deposition in the hippocampus by 50%. The phenolic compounds of pomegranate pulp may be responsible for these therapeutic effects [4], and the ethanolic extract was effective in doses of 800mg/kg/day [5].

The anti-inflammatory effects of pomegranate pulp extract were also observed in mouse models of AD, including a decrease in IL-6 and TNF-α in mouse brain samples [1,6]. This effect could contribute to attenuation of progression of the disease, once a prominent chronic inflammatory reaction is observed with the development of neurodegeneration. The isolated neuroprotective compounds were determined to be punicalagin and ellagic acid [1].

The long-term beneficial effects of pomegranate pulp extract were also observed in transgenic mice with AD that were treated for 15 months. Treated animals showed improvement in spatial memory and motor coordination and exhibited a decrease in anxiety behaviors, when compared to non-treated animals [7]. These reports collectively indicate that the pulp extract of the pomegranate is a promising therapy to hinder development of the neuropathological characteristics of AD.

The function of pomegranate pulp has been attributed to its antioxidant capacity given by phenolics, such as gallic acid, punicalagin α and punicalagin β, of which the latter two polyphenolics are unique to the pomegranate [8]. Punicalagin is a hydrolysable tannin (ellagitannin) with recognized antioxidant, anti-inflammatory and antiproliferative activities [9].

All previous studies described above were limited by studying the benefits of only the fruit pulp as neuroprotector. However, the bioactive properties of the pomegranate are not limited to its pulp. Several studies have shown that the peel, considered to be a leftover of processing by the food industry, has higher quantities of biologically active compounds in comparison to the edible pulp [10,11]. Thus, the aim of this study was to evaluate the neuroprotective effects of pomegranate peel extract (PPE) as a rich source of bioactive compounds to potentially delay the progression of dementia associated with AD. In support of our aim, we demonstrated the neuroprotective, anti-inflammatory and antioxidant properties of PPE.

## Materials and Methods

### Pomegranate samples

Pomegranate fruits of the *Wonderful* species that were used in this study were purchased from the Company of Warehouses and General Stores from the State of Sao Paulo (Companhia de Entrepostos e Armazens Gerais do Estado de São Paulo, CEAGESP), Brazil. The fruit was imported from the United States by the Othil Fruit Importer (São Paulo, SP, Brazil). The peel was separated from the pulp and seeds. The peel and pulp were stored in laminated polyethylene at 4°C for two days until use.

The extracts were prepared by percolation of the peel in an 80/20 hydroalcoholic solution at a 1:10 ratio (peel [g]: solvent [mL]) [12] on a shaker table (Model ET-1401, Tecnal, Piracicaba, SP, Brazil), protected from light and at room temperature (24 ± 2°C) for 24 hours. The 80:20 composition was chosen for use based on preliminary tests that demonstrated its higher antioxidant activity *in vitro* (data not shown) when compared to other concentrations of ethanol. The resulting extract was centrifuged at 1956.2g (Model NT 825, New Technique, Brazil), and the supernatant was concentrated at 35°C using a rotary evaporator (Model 801, Fisatom, Brazil) under vacuum, until completely dry and subsequently flash frozen in liquid nitrogen (White Martins, Piracicaba, Sao Paulo, Brazil). The packaged extracts were stored at -80°C for three days and protected from light until ready for microencapsulation.

### Chemical characterization of PPE

The amounts of bioactive compounds (phenolic compounds and flavonoids) from pomegranate peel extract were determined.

The content of the phenolic compounds (PC) in PPE was determined by the Folin–Ciocalteu colorimetric method [13] using gallic acid as standard (10–100 μg/mL concentration range). The amounts of PC were assayed in triplicate and expressed as mg of gallic acid in 100 g of PPE.

Quantification of the total flavonoids (FLA) was performed according to previous study [14], using catechin as a standard (0.020 to 0.10 mg/mL). The amounts of FLA were assayed in triplicate and expressed as mg of catechin in 100 g of PPE.

The quantification of total punicalagin in the peel extract was performed using high-performance liquid chromatography with an UV detector, according to the methodology described previously [15]. The injected sample volume was 1 μL and the quantification of punicalagin was carried out using calibration curves of the external reference standard containing 6 data points.

### Obtainment of microparticles

Microparticles were obtained by atomization, as described previously, using a LM-MSD 1.0 spray dryer (Labmaq, Brazil) [16]. Maltodextrin (Maltogill 20), a product obtained by the enzymatic conversion of yeast with dextrose degree 20, was used as the carrier agent. The carrier agent was added to the extract in a 1:1 proportion [16]. This mixture was maintained in a mechanical shaker (Fisaton, model 752, Sao Paulo, Brazil) at room temperature (23°C to 25°C) during the atomization process. The operational conditions used in the spray dryer were as follows: 0.7 mm diameter atomizer nozzle; 30 L/min drying air flow; 10 mL/min liquid flow; and 120 ± 10°C and 94 ± 1°C temperatures of air entrance and exit, respectively.

For the treatment of animals, microcapsules were diluted in filtered water in the concentration of 4.53 g/L immediatelly before use.

### Animals

Male C57Bl/6 mice of 3 months of age (average weight of 30 g) were maintained in individually ventilated cages (Alesco, Monte Mor, SP, Brasil), with food and water ad libitum. Room temperature was 24°C to 26°C and humidity was 55%.

Animals were divided into three experimental groups: Control (mice infused with vehicle and no oral treatment); βA (mice infused with amyloid-β peptide and no oral treatment); and βA + PPE (mice infused with amyloid-β and treated with pomegranate peel extract).

All efforts were made to reduce the number of animals and their suffering. The experimental proceedings were performed according to the “ethical principles for the use of laboratory animals” described by the Brazilian Society in Science of Laboratory Animals (SBCAL, Brazil). The experimental protocols were approved by the Animal Ethics Committee from Santa Casa de São Paulo School of Medical Sciences, under number 005/14.

### Induction of neurodegeneration with Aβ and treatment with pomegranate peel extract (PPE)

Chronic infusion with amyloid-β peptide was performed using mini-osmotic pumps (Alzet 1004, Cupertino, CA, EUA), following protocols described previously [17, 18, 19]. Briefly, the animals were injected with atropine (0.4 mg/kg, i.p.) 30 min before the beginning of surgery. They were then anesthetized with ketamine (0.035%, i.p.) and xylazine (0.035%, i.p.). The animal’s head was immobilized in stereotaxic equipment at coordinates: 0.8 mm anteroposterior and 1.0 mm mediolateral to bregma and 2.0 mm dorsoventral to cranium [20]. The other extremity of the cannula was attached to a polyvinylchloride catheter (Medical grade, OD = 1.14 mm, ID = 0.69 mm) that was connected to the mini-osmotic pump. The pumps were filled with vehicle ([4 mM HEPES, pH 7] + 0.22 nmol E-64 [Sigma #E3132]) for the control group or 0.51 nmol amyloid-β peptide 1–42 (Sigma #A9810) + 0.22 nmol E-64 for Aβ and Aβ + PPE groups. The pumps were subcutaneously implanted in the dorsum of the animal’s neck. The contents of the mini-osmotic pump were delivered at a flow of 0.11 μL/h for 28 days, using a total volume of 100 μL, according to the manufacturer’s guidelines [19].

Treatment with PPE began just after the end of surgery and recuperation. The dose used (800 mg/kg/day) was proportional to that described in a previous study [5].

### Behavioral Tests

The behavioral evaluation was done by a blinded examiner.

#### Evaluation of locomotor activity

Spontaneous locomotor activity was measured as described previously [18, 21] using an animal activity cage (model 7430, Ugo Basile, Comerio, Italy). The apparatus consisted of a transparent acrylic cage (35 cm x 23 cm x 20 cm) with a set of horizontal sensors to register locomotor activity and a set of vertical sensors to register rearing. Before and after the infusion period, each animal was placed alone inside the cage, and locomotion was immediately recorded for 5 min. During this period, immobility and grooming were also recorded.

#### Evaluation of spatial memory

Spatial memory was evaluated using the Barnes maze and a protocol based on previous studies [21, 22]. Briefly, the equipment consisted of a white arena (diameter 100 cm) positioned 1 m from the floor. The arena had 30 holes (5 cm diameter each) positioned radially and a black cardboard wall surrounding its circumference. The wall had four yellow figures to provide spatial orientation and a fluorescent lamp was placed above the center of the arena. The light created an uncomfortable environment to motivate the animals to escape from the arena into a dark box placed under one of the holes, which was filled with bedding material. In the training session, each mouse was placed in the center of the arena under a round acrylic box for 1 min. Then, the animal was released, and the time to find the escape box was recorded. The maximal time allowed for exploration was 5 min. If the animal did not find the correct hole, it was gently directed to the hole by the experimenter. Once inside the box, the animal was left there for 5 min. In the next 5 days (test days), twice a day, each mouse was placed inside the round acrylic box for 1 min and left to explore the arena until it found the escape box or for a maximum of 5 min. The escape box was always placed in the same hole for the same animal, but in different holes between animals. The maze was cleaned using a 5% ethanol solution before testing each animal. The initial dormancy and the number of errors during location of the escape box together with the distance travelled and the time spent in the quadrant of the escape box were assessed. Animal performance was recorded with a JVC Everio videocamera and analyzed with Smart Software, version 2.5.21 (Panlab Harvard Apparatus).

### Histological and biochemical analysis

After five weeks of treatment and behavioral tests, animals were anesthetized with isoflurane, and the brain was removed and immediately frozen in 2-dimethylbutane (-45°C to -55°C). Brains were stored at -80°C until use. Half of each brain was used for histological analysis. Samples were cut (20 μm) in a cryostat (−20°C to −22°C, Microm HM525, Germany), sections were mounted on gelatin-coated slides (Microscope Slides, 26x76 mm, K5-7105, Kasvi), and slides were stored at −80°C until use. The opposite brain hemisphere was used for ELISA.

For the ELISA, the frozen hemisphere was left to defrozen for a maximum of 1 min in order to permit the identification and isolation of the hippocampus and the cortex. Both areas were isolated and homogenized in lysis buffer containing 20 nM Tris-HCl (pH 8.0), 137 nM NaCl, 10% glycerol, 1% Triton X-100 and a tablet of protease inhibitors (Roche), in a total volume of 50 mL. The homogenates were centrifuged at 2973 g for 15 min at 4°C. Supernatants were collected and stored at -80°C.

Blood samples were collected in the absence of anticoagulant. After 30 min at room temperature, the samples were centrifuged at 2973 g for 15 min at 4°C for serum collection.

Livers were removed, immediately frozen in 2-dimethylbutane (-45°C to -50°C) and stored at -80°C until use.

#### Quantification of senile plaques

Six hemispheres from different mice of the Aβ-infused animals were analyzed. For each specimen, the senile plaques were measured in all sections obtained, that included areas from the frontal cortex until the most rostral part of the hippocampus. Frozen brain samples were warmed to room temperature (22°C) and were air-dried for 5–10 min. Quantification of senile plaques was performed as described previously [21]. Slides were washed 5 times in PBS and incubated in 0.1% Thioflavine S solution in PBS, containing 0.1% Triton, for 5 min. Then, sections were washed twice with PBS, incubated with 70% ethanol for 5 min and washed again 3 times with PBS. Finally, sections were covered with coverslips using Fluoroshield with DAPI (Sigma, USA). This entire process was done in a dark room. Analysis of senile plaques labeled with Thioflavine S was performed using an optic microscope (Leica M7000) with a suitable filter for fluorescence. Pictures of plaques were taken just after labeling, and plaques were counted manually and analyzed by dividing the number of plaques by the number of sections analyzed. This evaluation was done by a blinded examiner.

#### Quantification of total protein

The concentration of total protein in samples of brain homogenate supernatant and serum was evaluated using a colorimetric method [23].

#### Determination of BDNF density

To determine BDNF content in the cortex and hippocampus, the ELISA kit Emax® ImmunoAssay System was used (ref G7611, Promega, USA). The assay was performed according to the manufacturer’s instructions.

#### Evaluation of acetylcholinesterase (AChE) activity

To determine AChE activity in the cortex and in the hippocampus, the ELISA kit ABCAM® ImmunoAssay System, AB138871 was used. The assay was performed according to the manufacturer’s instructions. AChE activity was measured at 410 nm using Gen 5 2.0 software and a Biotek Eon spectrophotometer.

#### Lipid peroxidation

The extent of lipid peroxidation was determined by the concentration of thiobarbituric acid reactive substances (TBARS) [24, 25]. Liver homogenates or malondialdehyde (MDA) standards were pipetted into test tubes containing 20% (w/v) trichloroacetic acid (pH 3.5). The mixture was centrifuged at 743.46 g for 20 min at 4°C. The supernatant was collected and 2 mL of 0.67% (v/v) thiobarbituric acid was added to the sample. The samples were incubated at 95°C for 60 min, and the amount of MDA formed was measured spectrophotometrically at 532 nm and 660 nm. 1,1,3,3-Tetraethoxypropane (TEP), a form of MDA, was used as a standard in this assay (0 a 1.0068 μmol de TEP). TBARS concentration was expressed as nmol of malondialdehyde (MDA) per g of liver tissue.

#### Quantification of superoxide dismutase activity

The activity of superoxide dismutase enzyme (SOD) was evaluated in the serum and in cortex and hippocampus homogenates using a kit (Cayman, MI, USA) according to the manufacturer’s instructions. This kit utilizes tetrazolium salt for the detection of superoxide radicals generated by xanthine oxidase and hypoxanthine. One unit of SOD was defined as the amount of enzyme needed to produce 50% dismutation of superoxide radical. The SOD assay measures three types of SODs (Cu^2+^/Zn^2+^, Mn^2+^, and FeSOD).

#### Quantification of TNF-α

TNF-α levels in the serum, cortex and hippocampus homogenates were measured using the mouse TNF-α ELISA MAX Standard Kit (BioLegend) following the protocol provided by the manufacturer.

#### Quantification of hepatic lesion levels

Biomarkers for hepatic ischemic lesion, serum oxaloacetic glutamic transaminase (OGT) and serum glutamic pyruvic transaminase (GPT), were assessed using a commercial enzymatic and colorimetric assay provided by LaborLab (Sao Paulo, Brazil).

### Statistical analysis

Behavioral data obtained in the Barnes maze were expressed as the mean ± standard error and analyzed by repeated measures two-way ANOVA followed by Bonferroni’s multiple comparison test (days 1 to 4). The consolidation of memory was verified 29 days later, after the infusion with Aβ or vehicle and subsequent treatment. The performance of all groups on the 29th day was compared using one-way ANOVA followed by Tukey’s multiple comparison test.

Data related to all other experiments were analyzed by a Student t-test or one-way ANOVA followed by Tukey’s multiple comparison test or Bonferroni, when applicable.

All analyses were completed using Graph Pad Prisma 6.0 or SAS software (SAS, 2003). Values were considered significant when P < 0.05.

## Results and Discussion

### Chemical characterization of PPE

Pomegranate peel extract has a high amount of phenolic compounds (21.25 ± 0.16 mg of gallic acid/g) and flavonoids (7.60 ± 0.12 mg quercetin/g), which are important natural antioxidants. Punicalagin, the other phenolic compound that is the main bioactive compound present in the pomegranate, was in a concentration of 13.74 mg/g of extract.

### General observations

Pomegranate peel was administered as microparticles dissolved in water at a dose of 800 mg/kg/day. No significant difference (P = 0.13) was observed in water ingestion between pomegranate-treated and untreated groups (Ctrl: 47.65 ± 0.20 mL/week; Aβ: 47.76 ± 1.48 mL/week and Aβ + PPE: 48.61 ± 1.84 mL/week).

Toxicity was also monitored by mortality index (number of deaths/total number of animals), and no deaths were observed. At the end of the infusion period and treatment, body weights of animals infused with Aβ, whether treated or not treated with PPE (Aβ: 29.13 ± 0.35 g and Aβ + PPE: 28.05 ± 0.53 g), were similar to body weights of the control group (29.71 ± 0.54 g, P = 0.36). The same trend was observed when comparing liver weights, with no difference among the three groups (Ctrl: 1.69 ± 0.03 g; Aβ: 1.68 ± 0.03 g and Aβ + PPE: 1.61 ± 0.04 g, P = 0.67).

### Behavioral Tests

#### Evaluation of locomotor activity

**Fig 1:**
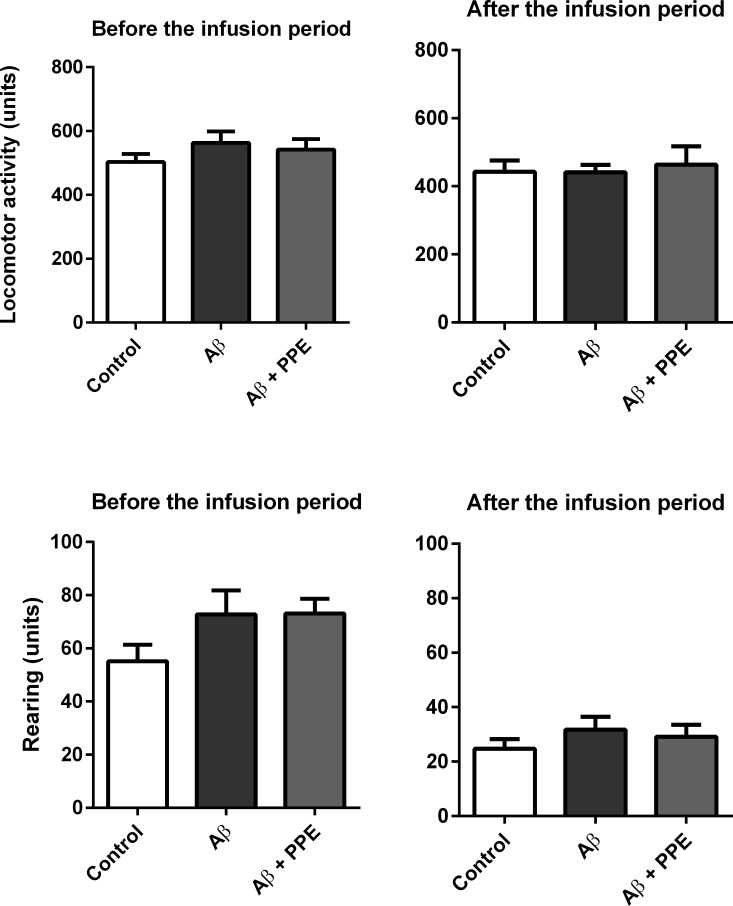
Infusion with amyloid-β peptide and treatment with pomegranate peel did not interfere with motor activity of the animals. There was no difference in the locomotor activity or the rearing between groups before or after the infusion period and treatment (Ctrl, n = 12; Aβ, n = 12 and Aβ + PPE, n = 12). Data are plotted as the mean ± S.E.M. of units of motor activity.

The behavioral tests used in this study depend on good locomotor activity, as animals need to explore the mazes and equipment for evaluation. In order to determine if the animals had any locomotor limitation before the experiments or any change in locomotor activity following treatment, all mice were subjected to the motor activity box. Infusion with Aβ and treatment with pomegranate extract did not alter the locomotor activity or rearing of animals in the beginning or at the end of the experiments (Fig 1).

At the end of the infusion period, all animals, independent of treatment, showed a reduction in rearing, but not in the locomotor activity, which is attributable to the animal’s habituation to the equipment as can be observed for some behavioral equipments [26].

#### Evaluation of spatial memory

Spatial memory and learning was evaluated using a Barnes maze. The hippocampus and some areas of the brain cortex are involved in spatial memory formation and recovery. Mice with hippocampal lesions have decreased performance in the Barnes maze [27]. Additionally, the hippocampus is one of the most compromised brain regions in patients with AD [28].

**Fig 2:**
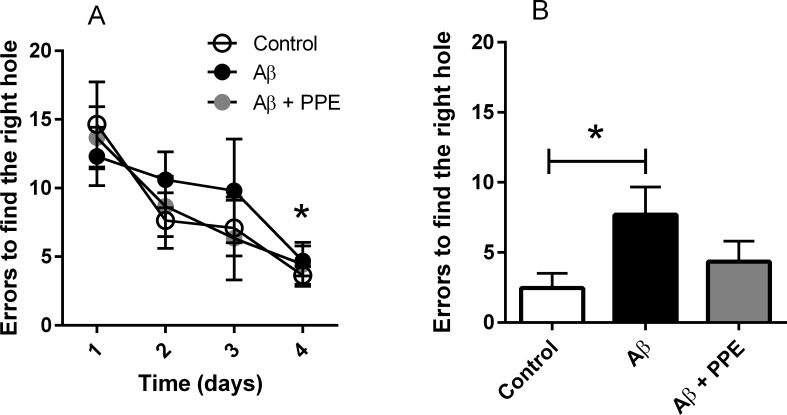
Treatment with pomegranate peel extract maintained spatial memory. In the “acquisition session”, all animals (Ctrl, n = 12; Aβ, n = 12 and Aβ + PPE, n = 12) learned the task as demonstrated by the significant decrease in number of errors to find the escape box on the 4^th^ day of exposition of all groups [F_(3,81)_ = 9.55, P < 0.0001, panel A].

The animals’ first exposure to the maze was considered the “acquisition session”. Animals were subjected to the equipment twice a day for 5 consecutive days. All animals, regardless of the group, showed a significant reduction [F_(3,81)_ = 9.55, P < 0.001] in the number of errors to find the escape box on the 1^st^ (Ctrl: 14.64 ± 3.10; Aβ: 12.30 ± 2.12 and Aβ + PPE: 13.67 ± 2.25) and 4^th^ days (Ctrl: 3.63 ± 0.65; Aβ: 4.70 ± 1.09 and Aβ + PPE: 4.44 ± 1.60), demonstrating that all animals had the capacity to learn the task (Fig 2A, 2B and 2C).

After the infusion period and treatment, the animals were subjected once again to the protocol (retest on day 29). Control and Aβ + PPE animals showed a similar number of errors to those observed before the infusion period (Fig 2B: Ctrl: 2.45 ± 1.05 errors; Aβ + PPE: 4.33 ± 1.48 errors), whereas the Aβ group showed a significant increase of 3.14 fold in the number of errors to find the escape box when compared to the Control group (7.70 ± 1.98, P < 0.05). These data suggest that treatment with pomegranate peel extract prevented the loss of memory observed in the untreated animals infused with Aβ.

It is possible that the decrease in memory, as determined by the increase in the number of errors to find the escape box in the Aβ group, is related to neurodegeneration in the cortex and hippocampus caused by chronic infusion with Aβ peptide [17,29] and formation of senile plaques in these brain areas [18].

There was no significant difference among the groups concerning other parameters observed during the test in the Barnes maze, including the initial time to find the escape box (P = 0.95), the total time (P = 0.73), the time spent in the right quadrant (P = 0.81) and the distance travelled (P = 0.09).

### Histopathological and biochemical analysis

#### Quantification of senile plaques

**Fig 3:**
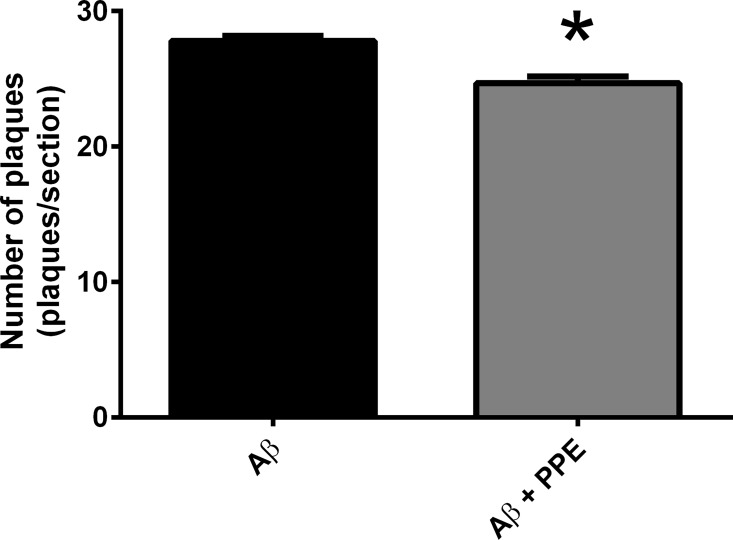
Treatment with pomegranate peel extract significantly decreased the number of plaques. Chronic infusion with Aβ peptide promoted the formation of senile plaques in the brain of mice, as described previously in rats [18]. The Aβ + PPE group (n = 6) presented a significant reduction in the number of plaques, when compared to the Aβ group (n = 6). Histograms and vertical bars are the means ± SEM. *: P < 0.01.

We previously showed that animals subjected to chronic infusion with Aβ peptide exhibited senile plaques as observed with Congo-Red staining [18]. In the present study, using a more stable and specific staining for senile plaques, thioflavine-S, a density of 27.77 plaques/sections was observed. Mice infused with Aβ and treated with PPE showed a significant reduction (11.2%) in the number of plaques (24.66 ± 0.52 plaques/sections, P < 0.01), when compared to Aβ infused animals (Fig 3). The reduction of plaque density can be linked to two specific inhibitors of β-secretase (BACE-1) present in the pomegranate peel, elagic acid (IC50 = 3.9 x 10–6 M) and punicalagin (IC50 = 4.1 x 10–7 M), or to the inhibition of y-secretase activity [30,31].

**Fig 4:**
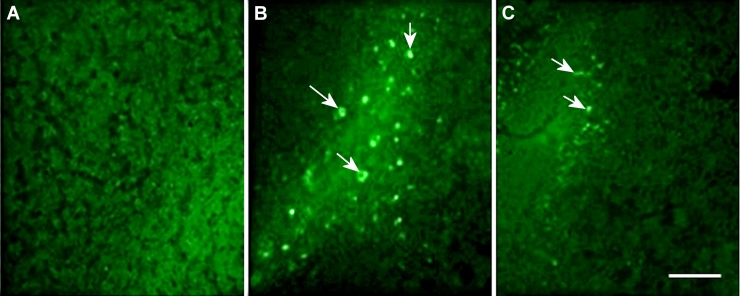
**Representative figures of thioflavine-S staining in brain of Ctrl (A), Aβ (B) and Aβ + PPE (C) groups.** The coronal sections showed are at approximately the same anatomical level (-1.58 mm from Bregma) and correspond to hippocampal areas. Representative figures are from samples run in the same batch during staining procedure. White arrows indicate the senile plaques. Scale bar is 140 μm.

These results reinforce the neuroprotective role of pomegranate peel extract with respect to characteristic neurodegeneration observed in AD patients. Inhibition of amyloid plaque formation is essential for memory maintenance and learning new tasks. Other functional foods that are rich in antioxidants and polyphenols also reduce the deposition of senile plaques in animals [32,33,34, 35,36]. Control mice (n = 6) did not have senile plaques (Fig 4).

#### Determination of BDNF density

BDNF plays an important role in the survival and function of dopaminergic and cholinergic neurons in the cortex and in the hippocampus [37,38,39,40]. Additionally, BDNF is a key molecule in synaptic plasticity, as it increases dendritic arborization and contact among neurons [41].

The depletion of neurotrophins increases the progression of dementia related to AD; thus, an increase in its expression can be a potential treatment for neurodegenerative diseases [42].

**Fig 5:**
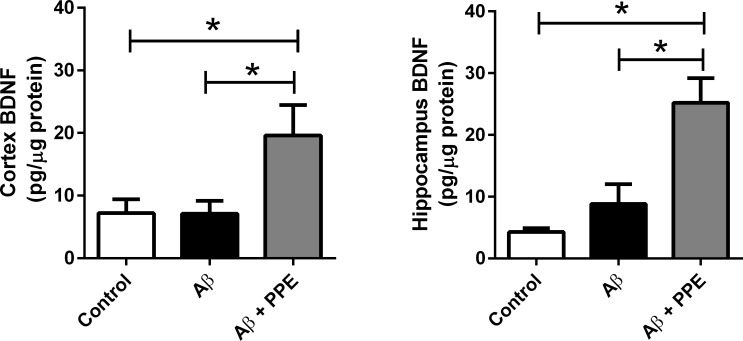
Treatment with pomegranate peel extract significantly increased BDNF levels. Control (n = 8) and Aβ groups (n = 8) did not have differences in the density of BDNF. However, treatment with PPE promoted a significant increase in BDNF in both the cortex and hippocampus on Aβ + PPE group (n = 8). Histograms and vertical bars are the means ± S.E.M. *: P < 0.05.

In our study, the Aβ group showed no significant difference in the level of BDNF in the cortex or in the hippocampus (7.12 ± 2.05 pg/μg and 8.80 ± 3.22 pg/ μg, respectively) when compared to those in the same areas of the control animals (7.23 ± 2.19 pg/μg and 4.27 ± 0.62 pg/μg, respectively). However, the Aβ + PPE group had a significant increase in the density of this neurotrophin, when compared to both control and Aβ groups, in the cortex (19.60 ± 4.87 pg/μg, P < 0.05) and in the hippocampus (25.21 ± 3.98 pg/μg, P < 0.05) (Fig 5). These data suggest that pomegranate peel extract increases the expression of BDNF, which can contribute to the neuroprotective action of this functional food during neurodegeneration.

Strategies to maintain or increase BDNF levels are widely studied. An increase in BDNF expression and consequent improvement in cognitive tasks was observed after treatment with phenolic compounds that are present in some foods, specifically fruits [43]. In addition, phenolic compounds present in the pomegranate peel may be involved in signaling pathways of neuroprotection, especially by increasing the expression of some neurotrophins that act via Trk and p75 receptors [42]. TrkA, TrkB and TrkC receptors have a high affinity for NGF, BDNF and NT 4/5 and NT-3, respectively [44], which are neurotrophic factors that may protect the brain from injury. These molecules are capable of reducing the progression of neurodegeneration, as they interact with Trk receptors and promote survival, growth, differentiation and maintenance of neuron [45].

The presence of gallic acid, rutine, quercetin and anthocyanins have been detected in pomegranate extract. These bioactive compounds can cross the blood-brain barrier and alleviate cognitive decline by inducing an increase in the expression of neurotrophins via TrkB signaling in the hippocampus [46].

#### Evaluation of acetylcholinesterase activity

Recently we showed that pomegranate peel extract has anticholinesterasic activity in vitro (personal publication). In the present study, this activity was verified in vivo.

**Fig 6:**
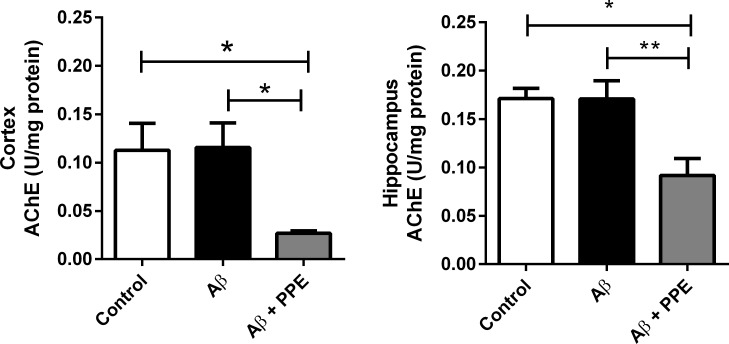
Treatment with pomegranate peel extract significantly decreased AChE levels. There was no significant difference in the activity of the AChE between the control and the Aβ groups. However, treatment with pomegranate promoted a significant reduction in the activity of this enzyme in both the cortex (Ctrl, n = 7; Aβ, n = 7 and Aβ + PPE, n = 8) and hippocampus (Ctrl, n = 6; Aβ, n = 7 and Aβ + PPE, n = 6). Data are the means ± S.E.M. *: P < 0.05; **: P < 0.01.

Following behavioral observations, mouse brains were extracted, and the activity of the acetylcholinesterase (AChE) enzyme was measured in the cortex and hippocampus. It was verified that the chronic infusion of Aβ did not change the activity of the AChE in the cortex (0.11 ± 0.06 U/mg protein) or in the hippocampus (0.17 ± 0.05 U/mg protein), when compared to the enzyme activity in the same areas of the vehicle-infused group (0.11 ± 0.07 U/mg protein and 0.17 ± 0.03 U/mg protein, respectively). This result was not expected, as many works have already shown that Aβ-treated animals normally present increases in AChE activity. However, in those works, the doses used were at least 32 times higher (0.159 nmol/μL to 2,0 nmol/ μL) than the dose used in the present work (0.005 nmol/μL) and the researchers made a single injection (or three single injections) of the Aβ (1–40 or 1–42), and not a chronic infusion [47,48,49,50,51]. In previous works, our research team observed neuron degeneration in the cortex and hippocampus and loss of memory in animals infused with 0.005 nmol/μL [18,19,29,52]. But in the present work, we verified that the observed loss of memory was not followed by increases in the AChE activity. Nevertheless, animals from the Aβ+PPE group had a significant reduction in the AChE activity in the cortex (47.0%, P < 0.05), when compared to the control group and when compared to the Aβ group (72.7%, P < 0.01, Fig 6).

In the same way, in the hippocampus, Aβ + PPE animals had a significant reduction of 47.0% in enzyme activity (0.09 ± 0.04 U/mg protein, P < 0.05) when compared to the Control group and a 47.0% reduction (P < 0.01) when compared to the Aβ group (Fig 6). These data reinforce the anticholinesterasic activity of pomegranate peel extract observed *in vitro*.

Acetylcholinesterase inhibitors (AChEIs) decrease the cholinergic deficits that lead to cognitive and neuropsychiatric dysfunction in patients with neurodegenerative diseases. Thus, inhibiting this enzyme in the brain is the main therapeutic target for delaying the development of dementia in AD patients. However, AChEIs may provoke parassympathomimetic side-effects, which may be unacceptable in aged patients. In this context, the search has begun for natural AChEIs with more efficient function and ease of procurement from natural resources, such as fruits [53, 54].

Many fruits contain bioactive substances that have anticholinesterasic activity, due to the presence of an elevated content of phenolic compounds that have this activity, including anthocyanins (delphinidin, pelargonidin and cyanidin), flavones (apigenin and luteolin) and flavonoids (quercetin, mircetin and kaempferol) [55,56,57]. Thus, it is possible that the anticholinesterasic activity of pomegranate peel extract is due to the presence of these compounds in the matrix [58].

#### Lipid peroxidation

Antioxidant activity was also evaluated in the liver of animals infused with Aβ and treated or not with pomegranate peel extract. The aim of MDA analysis was to verify the general antioxidant properties of the PPE, together with the SOD activity. As the brain samples were used in other analysis, there were no enough homogenates to evaluate the MDA contents in the brain. It was already described that the liver has greater amount of MDA as detected by the concentration of TBARS [25]. So, this tissue was used to verify if the chronic treatment with PPE could protect the organism from lipoperoxidation.

The quantification of malondialdehyde (MDA) has been reported as a good measure of the level of lipid peroxidation in biological samples [7,59].

Lipid peroxidation, probably caused by the generation of free radicals during the Aβ deposit, has been linked to the AD [60]. These oxidative events can lead to neuronal death and contribute to cognitive decline in patients with AD [61,62].

**Fig 7:**
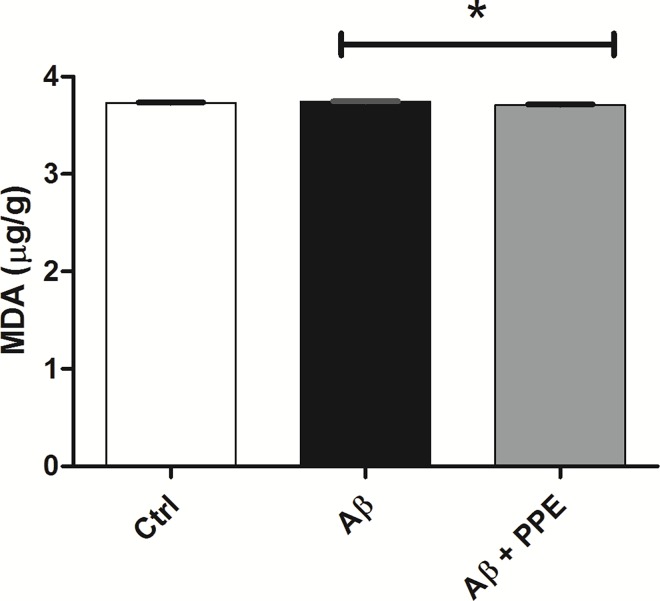
Treatment with pomegranate peel extract promoted a significant reduction in lipid peroxidation. There was no significant difference in the MDA content of the control (n = 9) and Aβ (n = 9) groups. Treatment with pomegranate peel extract induced a significant decrease in lipid peroxidation in Aβ + PPE (n = 9), when compared to the Aβ group. Histograms and vertical bars are the means ± S.E.M. *: P < 0.05.

In the present study, a slight increase in the quantity of MDA in the Aβ group (3.74 ± 0.005 μg MDA/g liver) was observed, when compared to the control group (3.73 ± 0.006 μg MDA/g liver), but with no significant difference, probably because of the short period of the Aβ infusion. However, treatment with pomegranate peel extract promoted a significant reduction in lipid peroxidation of the Aβ + PPE group (3.71 ± 0.006 μg MDA/g liver, P < 0.05), when compared to the Aβ group (Fig 7).

The intake of pomegranate peel extract could contribute to neuroprotection as an antioxidant and thereby stabilize or revert injury caused by oxidative stress. This antioxidant effect is also related to the high content of phenolic compounds (mainly punicalagin and gallic acid) in the extract [10].

Other studies previously described that the consumption of pomegranate (4% p/p in diet) for 15 months can reduce oxidative stress in transgenic mice for AD [7]. The proposed mechanism for antioxidant activity is the capacity of the extract to promote hydroxyl radical sink [63].

#### Activity of the antioxidant enzyme Superoxide Dismutase (SOD)

**Fig 8:**
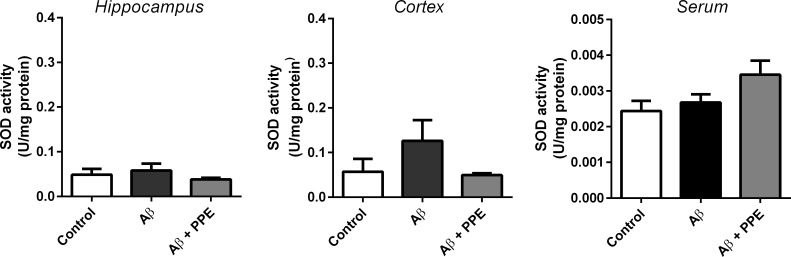
There was no significant change in the activity of superoxide dismutase (SOD) enzyme in the serum, cortex and the hippocampus of all animals. Brain homogenates and serum were tested to SOD activity by ELISA. The was no difference in SOD activity either in the hippocampus, cortex or serum of Control, Aβ and Aβ + PPE groups.

The activity of the antioxidant enzyme SOD were verified in the serum, cortex and hippocampus. Surprisingly, a non-significant increase in SOD activity was observed in the Aβ group cortex (0.13 ± 0.05 U/mg protein, n = 8) when compared to the Control group (0.06 ± 0.03 U/mg protein, n = 7). In the hippocampus or serum, no difference between the Control group (0.05 ± 0.01 U/mg protein, n = 7 and 0.0024 ± 0.0003 U/mg protein, n = 6, respectivelly) and the Aβ group (0.06 ± 0.01 U/mg protein, n = 7 and 0.0027 ± 0.0004 U/mg protein, n = 6) were observed. Moreover, the treatment of Aβ-infused animals with pomegranate peel extract did not change SOD activity significantly (hippocampus: 0.04 ± 0.01 U/mg protein, n = 7; cortex: 0.049 ± 0.001 U/mg protein, n = 8; serum: 0.0034 ± 0.0004 U/mg protein, n = 6, Fig 8).

These are contradictory and inconclusive outcomes, in that a reduction in SOD activity was predicted for chronic infusion with Aβ. Apart from the fact that the time of infusion could not be long enough to make a difference in SOD activity, the apparent increase is still intriguing.

Biochemical reports of antioxidant enzyme activity, such as SOD, in patients with AD are not consistent in the literature [64]. Many authors describe elevated activity of SOD in distinct brain areas [65,66,67], which was also observed in our study, whereas others did not notice changes in enzyme activity in the brain [68,69]. Additionally, other studies describe a reduction of 25 to 35% in the activity of SOD in the cortex, hippocampus and cerebellum of AD patients [70].

In this study, treatment with pomegranate peel extract promoted a reduction in lipid peroxidation in the liver, but did not increase the SOD acitivity in the brain. These data suggest that the antioxidant effect of the extract is independent of the endogenous antioxidant capacity.

#### Quantification of the cytokine TNF-α

**Fig 9:**
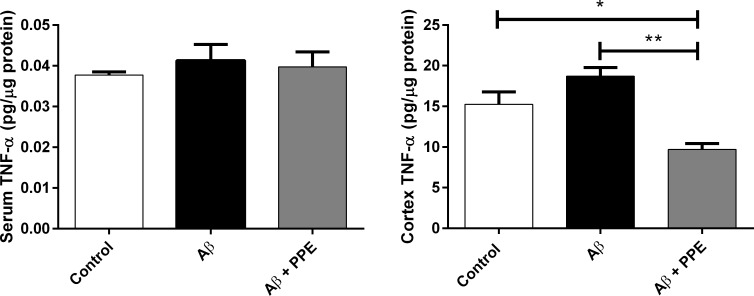
Infusion with amyloi-β increased the level of TNF-α in the brain and the treatment with pomegranate peel extract significantly decreased the inflammatory process. Brain homogenates and serum were tested to TNF-α by ELISA. There was no diference in TNF-α levels in the serum of Control (n = 5), Aβ (n = 6) and Aβ + PPE (n = 6) groups. In the cortex, the infusion of Aβ increased the TNF-α level. The treatment with Aβ + PPE (n = 6) decreased the TNF-α level when compared to the Control (n = 6) and Aβ (n = 5) groups. Histograms and vertical bars are means ± S.E.M. *: P < 0.05; **: P < 0.001.

Previous studies demonstrated that pomegranate extract has anti-inflammatory effects in experimental models [31]. Thus, we measured the quantity of inflammatory cytokine TNF-α in the cortex, serum and hippocampus. In the hippocampus, the level of the cytokine was undetectable (< 0.01 pg/μg protein). In the serum, the level was detectable in the three groups, but was also low and no difference among groups was observed (Ctrl: 0.038 ± 0.001 pg/μg protein, n = 5; Aβ: 0.041 ± 0.003 pg/μg protein, n = 6; Aβ + PPE: 0.039 ± 0.003 pg/μg protein, n = 6) (Fig 9).

Nevertheless, in the cortex, the presence of TNF-α was marked and much higher than in the serum, even for the control group. This finding may be due to the presence of the cannulae that was linked to the mini-osmotic pump. Moreover, a non-significant increase of 18.46% in the TNF-α content of the Aβ group was observed when compared to the control group (15.24 ± 1.53 pg/μg protein). This finding could be in response to the active inflammatory changes that follow senile plaques deposition in the brain after chronic infusion with Aβ [1,6] (Fig 9). In the Aβ+PPE group, however, a significant decrease in the TNF-α quantity of 36.29% (9.71 ± 0.72 pg/μg protein) was observed when compared to the control group (15.24± 1.53 pg/μg protein, P < 0.05) and of 48.05% when compared to the Aβ group (18.69 ± 1.07 pg/μg protein, P < 0.001) (Fig 9).

These data suggest that the compounds present in pomegranate peel extract may decrease the inflammatory process mediated by cytokines. According to some studies, the anti-inflammatory mechanism of the extract may include suppression of cyclooxygenase and lipoxygenase of the matrix [71,72]. The reduction in TNF-α content in the brain of transgenic mice for AD was verified with a dose of 6.25 mL/L of pulp extract. Treatment was maintained for three months [6]. Once more punicalagin and ellagic acid were responsible for the observed effects, as they inhibited the secretion of TNF-α.

#### Quantification of hepatic lesion index

**Fig 10:**
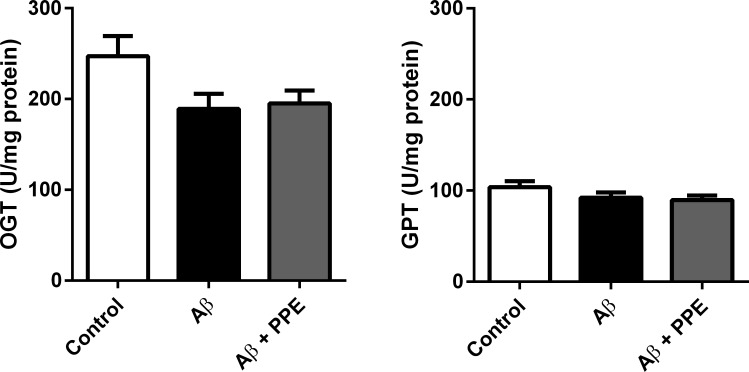
Intracerebroventricular infusion with amyloid-β peptide or consuption of pomegranate peel extract did not affect the hepatic function. Serum was tested to OGT and GPT by ELISA. There was no diference in the biomarkers levels in the serum of Control (n = 6), Aβ (n = 6) and Aβ + PPE (n = 6). Histograms and vertical bars are means ± S.E.M.

In order to verify a possible toxicological effect of pomegranate peel extract, biomarkers for hepatic ischemic damage oxaloacetic glutamic transaminase (OGT) and glutamic pyruvic transaminase (GPT) were evaluated in the animals’ serum. No difference in OGT (P = 0.089) or GPT (P = 0.19) was observed among the groups (Fig 10), which suggests that no hepatic lesion was produced with chronic i.c.v. infusion with Aβ or with treatment with pomegranate peel extract.

## Conclusions

Previous studies have shown that the pomegranate is a functional fruit with a variety of benefits for the central nervous system, creating a functional reserve that protects the brain from the neurodegenerative process that follows Alzheimer disease. In this study, we showed that pomegranate peel extract, a product that is normally considered as waste, is also a source of bioactive compounds that can reduce the accumulation of senile plaques, increase the expression of the neurotrophin BDNF, reduce the activity of the enzyme acetylcholinesterase, reduce lipid peroxidation and reduce the expression of the inflammatory cytokine TNF-α. Additionally, no toxicological effect in the liver was observed.

Taking together, these data support the concept that pomegranate peel extract can be considered a neuroprotector and could be used as an agent for treating Alzheimer's disease. This work is the first step for the clinical investigation of pomegranate peel extract in dementia.
